# Microbial Biogeography along the Gastrointestinal Tract Segments of Sympatric Subterranean Rodents (*Eospalax baileyi* and *Eospalax cansus*)

**DOI:** 10.3390/ani11113297

**Published:** 2021-11-18

**Authors:** Daoxin Liu, Jingyan Yan, Haijing Wang, Feng Jiang, Pengfei Song, Zhenyuan Cai, Tongzuo Zhang

**Affiliations:** 1 Key Laboratory of Adaptation and Evolution of Plateau Biota, Northwest Institute of Plateau Biology, Chinese Academy of Sciences, Xining 810001, China; liudx@qhu.edu.cn (D.L.); wanghj@nwipb.cas.cn (H.W.); jiangfeng@nwipb.cas.cn (F.J.); pfsong@nwipb.cas.cn (P.S.); caizhenyuan@nwipb.cas.cn (Z.C.); 2 University of Chinese Academy of Sciences, Beijing 100049, China; 3 College of Kunlun, Qinghai University, Xining 810016, China; 4 Qinghai Provincial Key Laboratory of Animal Ecological Genomics, Northwest Institute of Plateau Biology, Xining 810001, China; 5 College of Agriculture and Animal Husbandry, Qinghai University, Xining 810016, China; yanjingyanqh@163.com

**Keywords:** microbiota, gastrointestinal tract, source tracking, plateau zokor, Gansu zokor

## Abstract

**Simple Summary:**

The gut microbiota are crucial for hosts. For mammals, different gastrointestinal tract (GIT) segments have specific microbial communities, which play an essential role in the host’s nutrition, metabolism, immunity, and health. Plateau zokors (*Eospalax baileyi*) and Gansu zokors (*Eospalax cansus*) are closely related species that belong to the Spalacidae family, and are common pests in agriculture, forestry, and animal husbandry in northwestern China, with a sympatric distribution area in the transition zone between the Qinghai-Tibetan Plateau and the Loess Plateau. Here, the characteristics of the microbiota communities in different GIT segments of the plateau zokor and the Gansu zokor were studied, and the microbiota communities of the two zokor species were compared. Our results provide important information for further study on the function of microbiota communities in different GIT segments and the potential use of the gut microbiota as a new method for the population management of the zokors.

**Abstract:**

In this study, based on high-throughput sequencing technology, the biodiversity and the community structure of microbiota in different GIT segments (the stomach, small intestine, cecum and rectum) of plateau zokors and Gansu zokors were studied and compared. A source tracking analysis for the microbial communities of different GIT segments was carried out using the fast expectation–maximization microbial source tracking (FEAST) method. We found that, for both species, the microbial community richness and diversity of the small intestine were almost the lowest while those of the cecum were the highest among the four segments of the GIT. Beta diversity analyses revealed that the bacterial community structures of different GIT segments were significantly different. As for the comparison between species, the bacterial community compositions of the whole GIT, as well as for each segment, were all significantly different. Source tracking conducted on both zokors indicated that the soil has little effect on the bacterial community of the GIT. A fairly high percentage of rectum source for the bacterial community of the stomach indicated that both zokors may engage in coprophagy.

## 1. Introduction

Numerous studies have shown that gut microbiota play an essential role in host nutrition, metabolism, immunity and health [1,2]. Additionally, according to recent reports, the gut microbiota can even affect the cognitive performance of the host [3]. Thus, the host genome and the gut microbiome are considered to be a coadapted “hologenome” [4]. Many controlled experiment studies showed that the environmental factors such as diet, seasonal change, geographic location, population density, and captivity, can strongly affect the gut’s microbiota composition [5]. In addition, the host’s genetics were demonstrated to be another important factor influencing the gut microbiota [6,7,8]. However, the relative importance of the host’s genetics and the environment in shaping the gut microbiota continues to be a topic of great debate [9,10,11]. Analysis of the gut microbiota characteristics of sympatric and ecologically similar species may help to fill the knowledge gap.

In addition, although more and more studies focus on the gut microbiota of wildlife, because of sampling difficulties, the gastrointestinal tract (GIT) microbiota have generally been represented by the fecal microbiota in most studies on the wildlife [12,13,14,15]. In addition, studies on the characteristics and relationships of the microbial communities in different segments of the GIT are still very limited [16]. Exploring the microbial biogeography of wildlife will help us to understand the function of microbial communities—especially the adaptability of microbial communities to recalcitrant plant-based diets in natural environments [17].

Plateau zokors (*Eospalax baileyi*) and Gansu zokors (*Eospalax cansus*), belonging to the Spalacidae family in the order Rodentia, are closely related species endemic to China [18]. Both zokors are common pests in agriculture, forestry and animal husbandry in northwestern China and are mainly distributed in the Qinghai-Tibetan Plateau (QTP) and the Loess Plateau, respectively, with a sympatric distribution area in the transition zone between the two plateaus [19]. However, the distribution ranges of the two species and their sympatric area have not been studied nor defined clearly. As typical subterranean rodents, both zokors live underground for almost their entire life and mainly feed on the roots of plants [15,18]. The extreme living environment in the tunnel makes the zokor an ideal model to study the adaptation to hypoxia and high carbon dioxide concentrations [20,21]. Given the limitations imposed by underground burrows, zokors are faced with great challenges in food hunting, food digestion, and energy metabolism [15]. According to some reports, the plateau zokor exhibits acceptance and even preference for some common poisonous weeds [15], which makes the plateau zokor a good model to study the degradation of plant secondary metabolites. As we know, for hindgut-fermenting mammals, the fermentation of food is dependent on microbial communities in the GIT [17]. Hence, we speculated that studying the gut microbiota may help to explain the mechanism of the efficient use of limited food and the adaptation of the host to the tunnel environment. Furthermore, as closely related species with a sympatric distribution area, comparison of the gut microbiota communities of the plateau zokor and the Gansu zokor may help to evaluate the relative importance of the host genetics and the environment in shaping the gut microbiota. However, until now, the characteristics of the microbiota communities in different GIT segments of the plateau zokor and the Gansu zokor have not been reported, let alone the difference of the microbiota communities between the two species.

In this study, four plateau zokors and four Gansu zokors were captured in the sympatric area, which was first predicted by the maximum entropy (MaxEnt) model [22,23]. Then, the biodiversity and community structure of the microbiota in different GIT segments of the two zokor species were studied and compared using high-throughput sequencing technology. Because previous studies on gut microbial biogeography were mainly focused on the composition and diversity of microbiota across the GIT [16,24,25,26], and paid little attention to the relationship between the microbial communities of adjacent GIT parts, here, we carried out source tracking analysis of the microbial communities of different GIT segments, using the fast expectation–maximization microbial source tracking (FEAST) method [27]. The aim of our study was to answer the following questions: (1) What are the characteristics and relationships of the microbial communities in different GIT segments of the two zokor species? and (2) is there any difference between the gut microbiotas of the plateau zokor and Gansu zokor? If yes, what are the factors possibly responsible for the difference?

## 2. Materials and Methods

### 2.1. Habitat Suitability Analysis of the Plateau Zokor and the Gansu Zokor

#### 2.1.1. Data Collection on the Geographical Distribution of the Two Species

By consulting the literature from the past 10 years and through field investigation, geospatial data for both zokors were collected. After further screening and elimination (only one point was retained within the range of 1 km × 1 km), 225 data points for the plateau zokor and 72 data points for Gansu zokor remained.

#### 2.1.2. Climate Data

The climate variables were mainly obtained from the WorldClim website (http://www.worldclim.org/, accessed on 20 April 2020), including 19 bioclimatic variables, temperature (maximum, minimum, and average temperatures) and precipitation for each month. The resolution was 1 km. We used SPSS (version 26) to remove the environmental variables with high correlations (Pearson correlation coefficients of |r| ≥ 0.80).

#### 2.1.3. Habitat Suitability Analysis of the Two Species Based on the MaxEnt Model

The optimal model parameters were combinations of linear, quadratic, product, threshold, and hinge features, and a regularization multiplier (β = 1). Then, the collected data were randomly grouped using 25% of the test set and 75% of the training set according to the model description. In addition, the jackknifing method was used to evaluate the contribution rate of the environmental factor, and there were 10 repetitions. An area with a distribution probability of ≥0.2 was identified as suitable habitat.

### 2.2. Samples Collection

To minimize the impact of the geographic location and other environmental conditions, based on the result of the habitat suitability analysis in Section 2.1, we designed the sample collection in Huzhu county, Haidong city, Qinghai province, a sympatric distribution area of the two species, which was revealed in the habitat suitability analysis and demonstrated by our field investigation. The plateau zokor and the Gansu zokor were, respectively, sampled in Donggou township (36°53′42.3″ N, 102°07′8.1″ E, with an altitude of 3014 m) and Songduo township (36°41′42.8″ N, 102° 11′51.8″ E, with an altitude of 2896 m), which is 22.89 km away from the former site, in May 2020. For each zokor species, 4 male adults were captured with live traps. After anesthesia with diethyl ether, the zokors were killed by cervical dislocation, and then for each zokor, the stomach contents, small intestine contents, cecal contents and rectal contents were separately collected and stored immediately in liquid nitrogen, as well as the soil sample from the tunnel.

### 2.3. DNA Extraction and Illumina MiSeq Sequencing

Total DNA was extracted from 40 samples using the E.Z.N.A.^®^ soil DNA Kit (Omega BioTek, Norcross, GA, USA), according to the manufacturer’s protocol. All DNA samples were quality checked and the concentration was quantified by Nanodrop 2000 spectrophotometers (Thermo Fisher Scientific, Wilmington, DE, USA). Bacterial 16S rRNA gene fragments (V3-V4) were amplified from the extracted DNA using primers 338F (5′-ACTCCTACGGGAGGCAGCAG-3′) and 806R (5′-GGACTACHVGGGTWTCTAAT-3′) [28], and the following PCR conditions: 30 s at 95 °C, 30 s at 55 °C, and 45 s at 72 °C for 27 cycles. PCRs were performed with 4 μL of 5× TransStart FastPfu buffer (Thermo Fisher Scientific, Wilmington, DE, USA), 2 μL of 2.5 mM deoxynucleoside triphosphates (dNTPs), 0.8 μL of each primer (5 μM), 0.4 μL of TransStart FastPfu DNA Polymerase (Thermo Fisher Scientific, Wilmington, DE, USA), 10 ng of extracted DNA, and finally, ddH_2_O to make up 20 μL. Agarose gel electrophoresis was performed to verify the size of the amplicons. The amplicons were subjected to paired-end sequencing on the Illumina MiSeq sequencing platform using PE300 chemical at Majorbio Bio-Pharm Technology Co. Ltd. (Shanghai, China). The Sequence data are available from the Sequence Read Archive (SRA) BioProject PRJNA749684.

### 2.4. Amplicon Sequence Processing and Analysis

The bioinformatics pipeline was mainly conducted in QIIME2-2020.2 [29]. Briefly, after being demultiplexed, the resulting sequences were merged with FLASH (v1.2.11) [30] and quality filtered with fastp (0.19.6) [31]. Then, DADA2 [32] were adopted (via q2-dada2) to denoise the sequences with recommended parameters, which include cutting low quality nucleotides, filtering out noisy sequences, chimeric sequences and singletons, joining denoised paired-end reads, and dereplicating, to obtain a raw amplicon sequence variants (ASVs) table and raw ASVs representative sequences.

The reference sequence annotation and curation pipeline (RESCRIPt) was used to prepare a QIIME2 compatible Naive Bayes classifiers based on the curated the silva NR99 (version 138) database, following the protocol suggested by the author (https://forum.qiime2.org/t/processing-filtering-and-evaluating-the-silva-database-and-other-reference-sequence-data-with-rescript/15494, accessed on 13 May 2021). Taxonomy classification was done via Q2-feature-classifier classify-sklearn after sequences with a confidence less than 0.8 filtered. The taxonomy-based filtering was applied to remove all ASVs that contain mitochondria, chloroplast, or archaea.

Raw ASVs table were then normalized by a rarefaction to the minimum sequencing depth of all samples (depth = 31,565) to remove sample heterogeneity for all further analyses.

We used q2-diversity to construct an alpha rarefaction curve (iterations = 999, steps = 10) on observed ASVs to determine if the richness of all samples has been fully observed. Alpha diversity indices, including Chao1, Observed ASV, and Shannon metric, were calculated in QIIME2 using the diversity plugin. Alpha diversities were compared by Kurskal–Wallis pairwise test between different sample groups using qiime diversity alpha-group-significance. All distance-based analyses were performed using the q2-diversity-lib plugin based on the respective Bray–Curtis distances. Principal Coordinate Analysis (PCoA) based on Bray–Curtis distances and permutational multivariate analysis of variance (PERMANOVA) were performed using the package vegan and visualized using the package ggplot2 in R and Rstudio [33]. STAMP [34] was used for comparing the differences between the groups in different categories.

Fast expectation–maximization microbial source tracking (FEAST) was designed to unravel the origins of microbial communities. In this study, microbial communities of one GIT segment were considered as a sink, and the soil and the upstream segment were considered to be the two major known sources, while the rectum was considered as the upstream segment of the stomach. Taxa that could not map to the input sources were categorized as the unknown. Majorbio Co., Ltd., was commissioned to complete all experiments described in the sections on DNA extraction and Illumina MiSeq sequencing. The analyses of the 16S rRNA microbiome sequencing data (except source tracking) were performed using the free online Majorbio Cloud Platform (www.majorbio.com, accessed on 12 June 2021).

## 3. Results

### 3.1. Habitat Suitability Analysis of the Plateau Zokor and the Gansu Zokor

**Figure 1:**
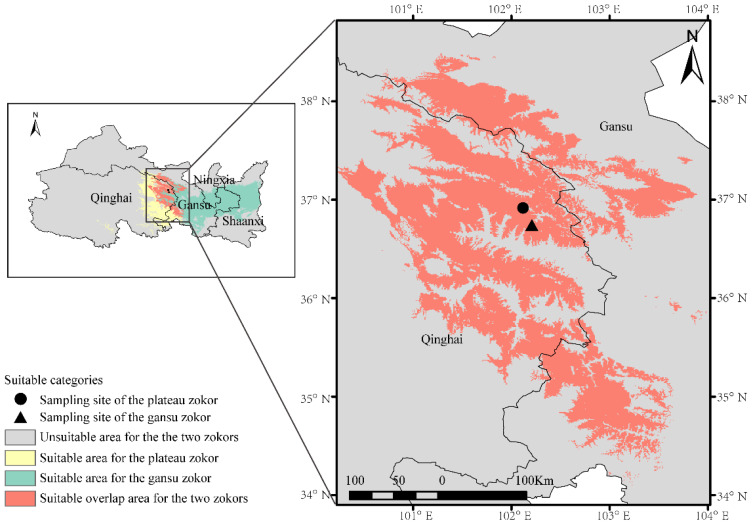
Habitat suitability analysis and the sampling sites of the plateau zokor and the Gansu zokor.

The results of the model prediction show that the average area under the ROC curve (AUC) values of the two zokor species were 0.988 ± 0.000 and 0.988 ± 0.001, respectively, which indicates that the model prediction results of habitat suitability for the plateau zokor and the Gansu zokor were highly reliable, because the AUC value of each species exceeded 0.9. As shown in Figure 1, the suitable habitat of the plateau zokor is mainly concentrated in eastern Qinghai and in the southern Hexi Corridor in Gansu, while the Gansu zokor is mainly distributed in eastern Qinghai, the southern Hexi Corridor in Gansu, southern Ningxia and central Shaanxi, accounting for 8.5% and18.2% of the total area, respectively. The two species overlap in the area at the junction of eastern Qinghai and Gansu, and the overlap area is 63,797 km^2^, accounting for 3.2% of the total area.

Elevation and bio12 (annual precipitation) were, respectively, determined as the important factor affecting species distribution according to the corresponding curves of the plateau zokor and the Gansu zokor.

### 3.2. Sequencing Metrics

**Figure A1:**
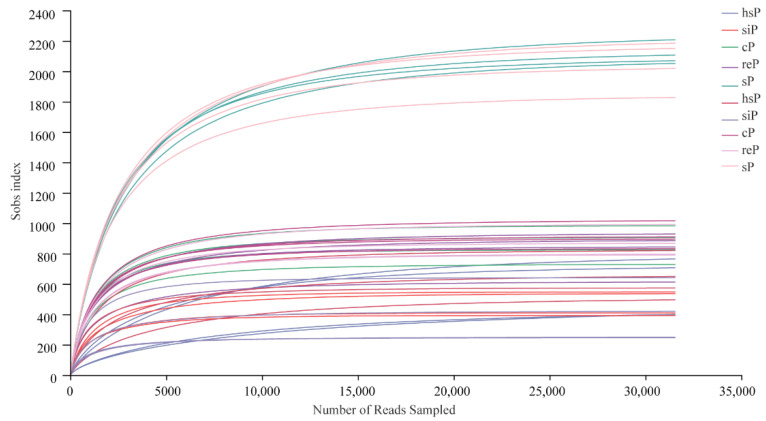
The rarefaction curves. The number of observed ASVs gradually plateaued as the sequencing depth increased, which demonstrates that each sample had sufficient ASVs to reflect the maximum level of bacterial diversity.

A total of 4,611,860 × 2 reads of raw data were obtained, and the average number of reads per sample was 103,586 ± 17,879. After quality filtering, 1,919,964 qualified reads with an average length of 411.95 bp were included in the clean data. To standardize the efforts across the samples, each sample was rarefied to 31,565 sequences. After being denoised using DADA2, for the plateau zokor, 4319 and 4774 ASVs were detected in the GIT and the tunnel soil, respectively, with only 101 shared ASVs, and for the Gansu zokor, 3646 and 4568 ASVs were detected in the GIT and the tunnel soil, respectively, with only 28 shared ASVs, resulting in a total of 15,046 ASVs harvested from all 40 samples (Table 1). The rarefaction curves of the Sobs index (the quantity of observed ASVs) gradually plateaued as the sequencing depth increased, which demonstrated that each sample had sufficient ASVs to reflect the maximum level of bacterial diversity, and almost all the present bacterial species were detected at the present sequencing depth. Judging from the gradual horizontalization of the rarefaction curve and rank abundance (see Figure A1), the sample size and sequencing depth were adequate.

### 3.3. Alpha Diversity of the Bacterial Community in the Two Zokor Species’ GITs

**Figure 2:**
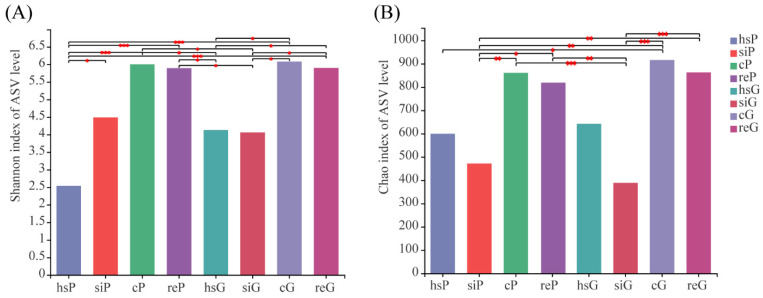
Alpha diversity analysis: (**A**) Shannon index and (**B**) Chao index. * 0.01 < *p* ≤ 0.05; ** 0.001 < *p* ≤ 0.01; *** *p* ≤ 0.001. P represents the plateau zokor and G represents the Gansu zokor. The stomach, small intestine, cecum, and rectum of the plateau zokor (the Gansu zokor) are labeled as hsP, siP, cP, and reP (hsG, siG, cG, and reG), respectively.

The Alpha-diversity indices (Shannon and Chao1) were used for the analysis of species diversity of the bacterial community in different segments of the GIT and the tunnel soil (Figure 2). For both the plateau zokor and Gansu zokor, the ranking of the bacterial community diversity of the different segments measured by the two indices (Shannon and Chao1) is similar. The bacterial Community diversity of the tunnel soil was higher than every GIT segment. Among the different GIT segments, the bacterial community diversity of the cecum was the highest, then the rectum, and the stomach and the small intestine were last. There were no significant differences in the bacterial community diversity for any GIT segment between the two species.

### 3.4. Beta Diversity of the Bacterial Community in the Two Zokor Species’ GITs

**Figure 3:**
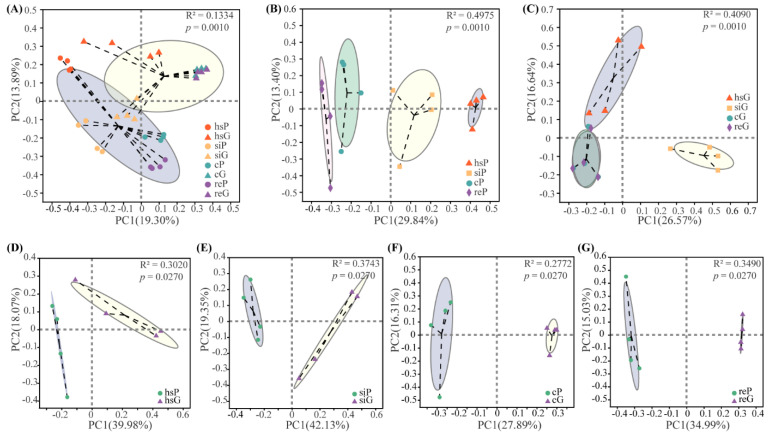
Bray–Curtis distance–based principal coordinate analysis (PCoA). P represents the plateau zokor and G represents the Gansu zokor. The bacterial community of the stomach, small intestine, cecum, and rectum of the plateau zokor (the Gansu zokor) are labeled as hsP, siP, cP and reP (hsG, siG, cG and reG). (**A**) PCoA analysis of the whole GITs of plateau and Gansu zokors. (**B**) PCoA analysis of different GIT segments of plateau zokors. (**C**) PCoA analysis of different GIT segments of Gansu zokors. (**D**) PCoA analysis of hsP vs hsG. (**E**) PCoA analysis of siP vs siG. (**F**) PCoA analysis of cP vs cG. (**G**) PCoA analysis of reP vs reG.

PCoA was used to evaluate the difference of the bacterial community structure between two zokor species and across different gut regions based on Bray–Curtis distance metrics (Figure 3). Between species, the bacterial communities of the whole GIT (Adonis R^2^ = 0.133, *p* = 0.001), the stomach (Adonis R^2^ = 0.302, *p* = 0.027), the small intestine (Adonis R^2^ = 0.374, *p* = 0.027), the cecum (Adonis R^2^ = 0.277, *p* = 0.027) and the rectum (Adonis R^2^ = 0.349, *p* = 0.027) all showed significant differences. Additionally, for both the plateau and Gansu zokors, the bacterial communities of different gut regions were also significantly different (Adonis R^2^ = 0.498, *p* = 0.001; R^2^ = 0.409, *p* = 0.001, respectively).

### 3.5. Microbiome Composition from the GITs of Zokors and Soil

In total, all 15,046 identified ASVs from 40 samples were sorted into 39 phyla, 117 classes, 271 orders, 429 families and 872 genera. The numbers of the microbiological taxonomic units at different levels from the GITs of the two species are shown in Table 1.

**Figure 4:**
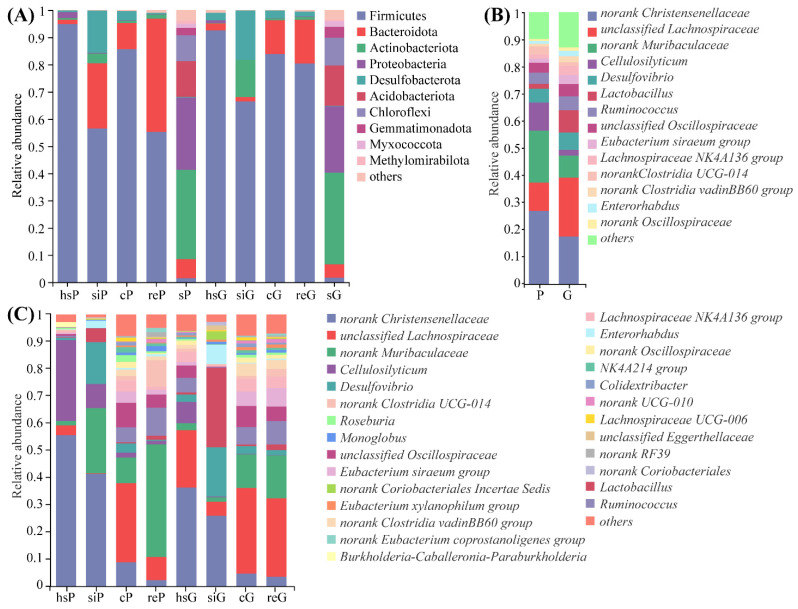
Relative abundance histograms at the phylum level (**A**). Relative abundance histograms at the genus level for plateau and Gansu zokors (**B**). Relative abundance histograms at the genus level for different GIT segments of plateau and Gansu zokors (**C**). “Others” represents the phyla with a relative abundance of less than 1%. P represents the plateau zokor and G represents the Gansu zokor. The stomach, small intestine, cecum, rectum, and tunnel soil of the plateau zokor (the Gansu zokor) are labeled as hsP, siP, cP, reP, and sP (hsG, siG, cG, reG, and sG), respectively.

The relative abundances at the phylum level for different GIT segments and the tunnel soil of the two species are shown in Figure 4. Firmicutes was the predominant phylum in every GIT segment of the two species. The highest relative abundance of Firmicutes (94.87%) was detected in the stomach of the plateau zokor (hsP), while the rectum of the plateau zokor (reP) had the lowest relative abundance (55.17%).

In the plateau zokors, the predominant phyla (mean relative abundance of >1%) in different GIT segments were different, and consisted of Firmicutes (94.87%), Proteobacteria (2.20%), and Bacteroidota (1.67%) in stomach (hsP), Firmicutes (56.43%), Bacteroidota (23.98%), Desulfobacterota (15.46%), and Actinobacteriota (3.63%) in the small intestine (siP), Firmicutes (85.66%), Bacteroidota (9.62%), and Desulfobacterota (3.43%) in the cecum (cP), and Firmicutes (55.17%), Bacteroidota (41.68%), and Actinobacteriota (1.46%) in the rectum (reP). In the tunnel soil of the plateau zokor (sP), nine dominant phyla (mean relative abundance of >1%) were identified, which were Actinobacteriota (32.73%), Proteobacteria (26.48%), Acidobacteriota (13.21%), Chloroflexi (9.61%), Bacteroidota (6.94%), Gemmatimonadota (2.67%), Myxococcota (1.50%), Firmicutes (1.48%), and Methylomirabilota (1.17%).

In Gansu zokors, the predominant phyla (mean relative abundance of > 1%) in different GIT segments were different, and consisted of Firmicutes (92.97%), Desulfobacterota (2.70%), and Bacteroidota (2.36%) in the stomach (hsG), Firmicutes (70.77%), Desulfobacterota (16.08%), Actinobacteriota (11.48%), and Bacteroidota (1.55%) in the small intestine (siG), Firmicutes (83.89%), Bacteroidota (12.20%) and Desulfobacterota (3.03%) in the cecum (cG), Firmicutes (78.37%), Bacteroidota (18.39%), Desulfobacterota (1.51%), and Actinobacteriota (1.00%) in the rectum (reG). In the tunnel soil of the Gansu zokor (sG), eight dominant phyla (mean relative abundance of > 1%) were identified, which were Actinobacteriota (34.35%), Proteobacteria (23.53%), Acidobacteriota (15.21%), Chloroflexi (10.69%), Bacteroidota (4.48%), Gemmatimonadota (3.97%), Myxococcota (1.93%), and Firmicutes (1.59%).

**Figure A2:**
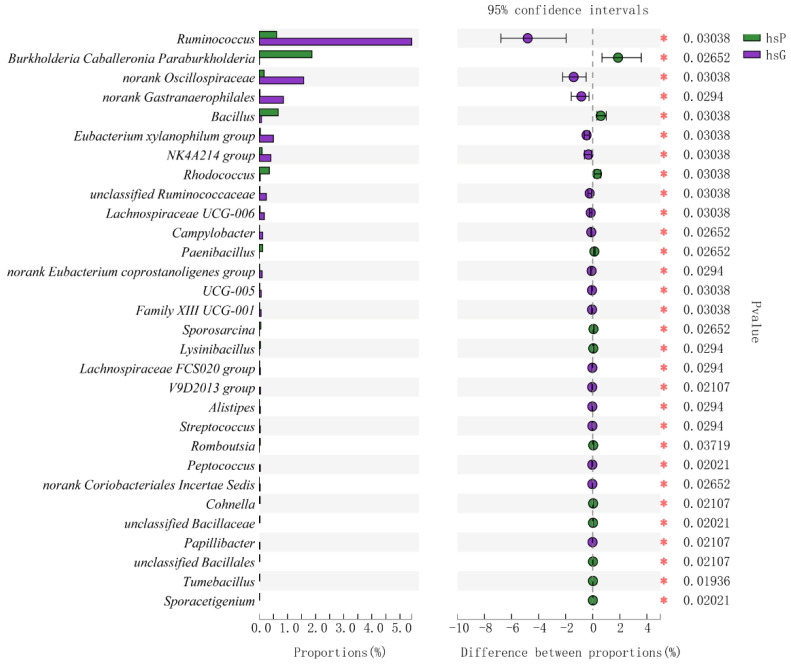
Wilcoxon rank–sum test bar plot at the genus level for the comparison of the microbiota in the stomach. The relative abundances of 30 genera were tested and found to be significantly different between the hsP and hsG groups. * 0.01 < *p* < 0.05.

**Figure A3:**
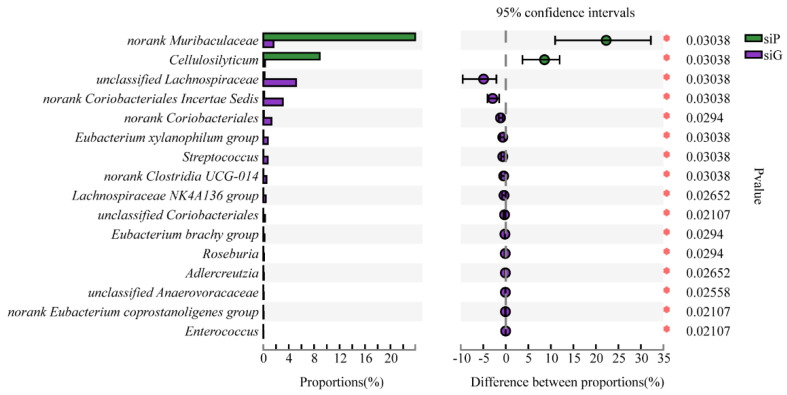
Wilcoxon rank–sum test bar plot at the genus level for the comparison of the microbiota in the small intestine. The relative abundances of 16 genera were tested and found to be significantly different between the siP and siG groups. * 0.01 < *p* < 0.05.

**Figure A4:**
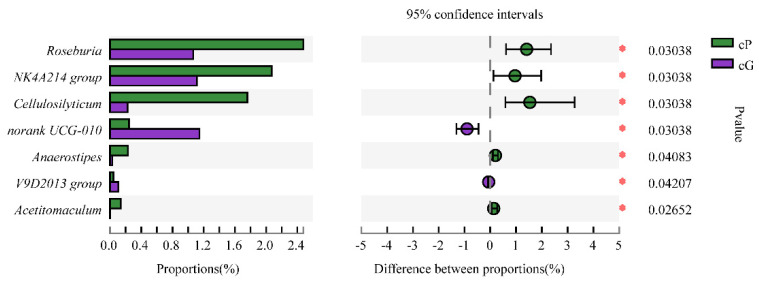
Wilcoxon rank–sum test bar plot at the genus level for the comparison of the microbiota in the cecum. The relative abundances of seven genera were tested and found to be significantly different between the cP and cG groups. * 0.01 < *p* < 0.05.

**Figure A5:**
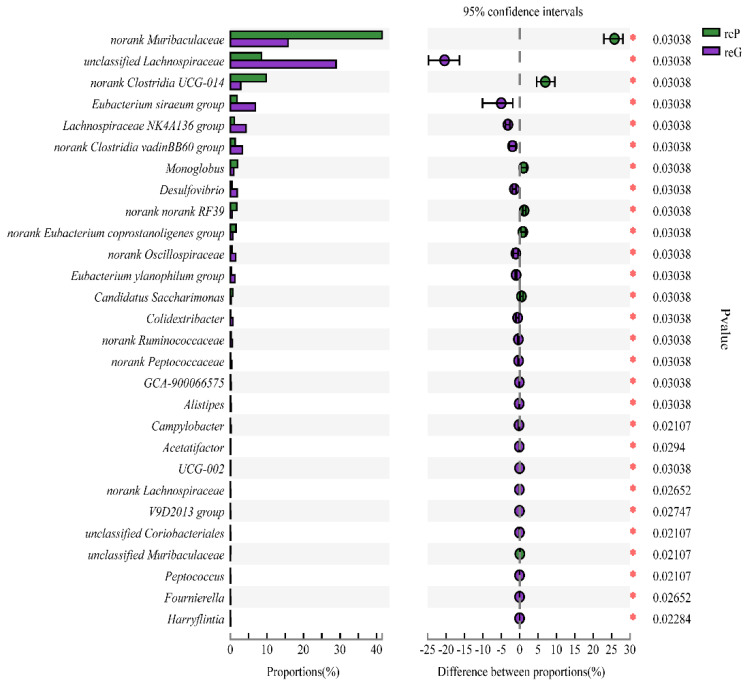
Wilcoxon rank–sum test bar plot at the genus level for the comparison of the microbiota in the rectum. The relative abundances of 28 genera were tested and found to be significantly different between the reP and reG groups. * 0.01 < *p* < 0.05.

At the genus level, the relative abundances of 30 genera in the stomach, 16 genera in the small intestine, seven genera in the cecum and 28 genera in the rectum, were revealed to be significantly different between the plateau and Gansu zokors. The results are shown in Figure A2, Figure A3, Figure A4 and Figure A5. The relative abundances at the genus level for the whole GIT of the two species are shown in Figure 4B. In this study, the genera shared by both/all compared groups and with the relative abundances for both/all groups higher than 1% are considered as the core microbiota. For the whole GITs of plateau and Gansu zokors, 11 genera were identified as the core microbiota, which are *norank Christensenellaceae* (27.0% in P and 17.6% in G), *norank Muribaculaceae* (19.1% in P and 8.1% in G), *unclassified Lachnospiraceae* (10.4% in P and 21.7% in G), *Desulfovibrio* (5.0% in P and 6.4% in G), *Ruminococcus* (4.1% in P and 5.1% in G), *unclassified Oscillospiraceae* (3.6% in P and 4.5% in G), *norank Clostridia UCG-014* (3.0% in P and 1.4% in G), *Lactobacillus* (1.8% in P and 8.1% in G), *Eubacterium siraeum group* (1.6% in P and 3.4% in G), *Lachnospiraceae NK4A136 group* (1.4% in P and 3.3% in G), and *Enterorhabdus* (1.1% in P and 2.0% in G).

The relative abundances at the genus level for different GIT segments of the two species are shown in Figure 4C. The relative abundances of two genera (*norank Christensenellaceae* and *norank Muribaculaceae*) for plateau zokors and four genera (*norank Christensenellaceae*, *unclassified Lachnospiraceae*, *norank Muribaculaceae*, and *Desulfovibrio*) for Gansu zokors were higher than 1% in all the four GIT segments. For different GIT segments of plateau zokors, the numbers of the genera with a relative abundance higher than 1% are, respectively, five for the stomach with *norank Christensenellaceae* (55.7%), *Cellulosilyticum* (29.7%), *unclassified Lachnospiraceae* (3.6%), *Burkholderia-Caballeronia-Paraburkholderia* (1.9%), and *norank Muribaculaceae* (1.6%) as the top five genera, six for the small intestine with *norank Christensenellaceae* (41.4%), *norank Muribaculaceae* (23.9%), *Desulfovibrio* (15.5%), *Cellulosilyticum* (8.9%), and *Lactobacillus* (5.1%) as the top five genera, 16 for the cecum with *unclassified Lachnospiraceae* (29.2%), *norank Muribaculaceae* (9.4%), *unclassified Oscillospiraceae* (9.0%), *norank Christensenellaceae* (8.8%), and *Ruminococcus* (5.5%) as the top five genera, and 16 for the rectum with *norank Muribaculaceae* (41.5%), *Ruminococcus* (10.3%), *norank Clostridia UCG-014* (9.8%), *unclassified Lachnospiraceae* (8.5%), and *unclassified Oscillospiraceae* (4.9%) as the top five genera. For different GIT segments of Gansu zokors, the numbers of the genera with a relative abundance higher than 1% are, respectively, 12 for the stomach with *norank Christensenellaceae* (36.4%), *unclassified Lachnospiraceae* (21.1%), *Cellulosilyticum* (7.8%), *Ruminococcus* (5.4%), and *unclassified Oscillospiraceae* (4.6%) as the top five genera, nine for the small intestine with *Lactobacillus* (29.2%), *norank Christensenellaceae* (25.9%), *Desulfovibrio* (18.1%), *Enterorhabdus* (7.1%), and *unclassified Lachnospiraceae* (5.2%) as the top five genera, 16 for the cecum with *unclassified Lachnospiraceae* (31.5%), *norank Muribaculaceae* (12.4%), *un-classified Oscillospiraceae* (7.8%), *Ruminococcus* (6.3%), and *Eubacterium siraeum group* (5.3%) as the top five genera, and 16 for the rectum with *unclassified Lachnospiraceae* (28.9%), *norank Muribaculaceae* (15.8%), *Ruminococcus* (8.6%), *Eubacterium siraeum group* (6.8%), and *unclassified Oscillospiraceae* (5.3%) as the top five genera.

### 3.6. Microbial Source Tracking of the Different Segments in the Two Zokor Species’ GITs

**Figure 5:**
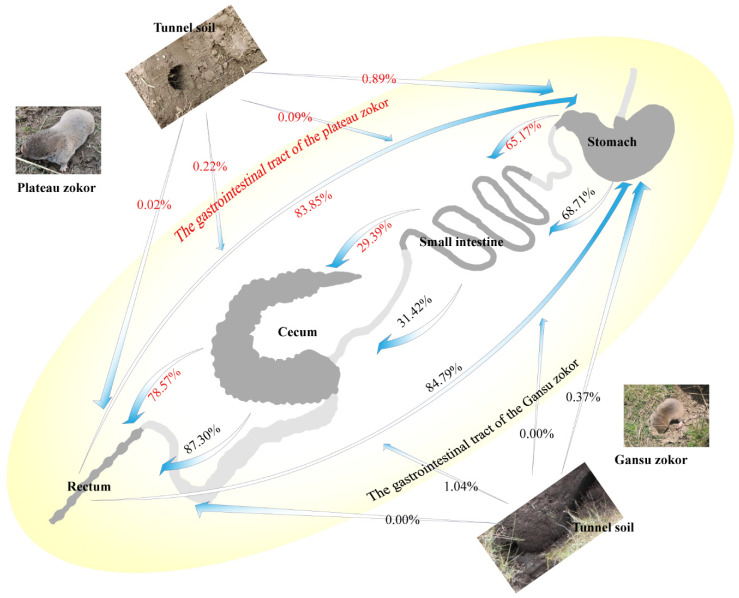
Fast expectation–maximization microbial source tracking (FEAST) analysis. Percentages for the plateau zokor are shown in red, and percentages for the Gansu zokor are shown in black. The values one near to the arrows are the percentages of the microbiota contributed by the GIT segment behind the arrow to the GIT segment in front of the arrow.

By FEAST analysis (Figure 5), we found that the soil contributed little to the microbiota of the GITs of the two species, and only the cecum of the Gansu zokor (cG) obtained more than 1% of the ASVs from the soil, which was 1.04%. The stomach was the dominant contributor to the microbiota of the small intestine, and 65.17% in the plateau zokor and 68.71% in the Gansu zokor of the microbiota of the small intestinal was estimated to have originated from the stomach. The highest microbiota percentages from “unknown” sources were found in the cecum, with 70.39% in the plateau zokor and 67.54% in the Gansu zokor. The cecum was the dominant contributor to the microbiota of the rectum, accounting for 78.57% in the plateau zokor and 87.30% in the Gansu zokor. Interestingly, the rectum was recognized as the dominant contributor to the microbiota of the stomach; 83.85% in the plateau zokor and 84.79% in the Gansu zokor of the stomach microbiota was estimated to have come from the rectum.

As revealed by the *t* test, for certain segments of the GIT, the percentages of the microbiota originating from a certain source are similar between the plateau and Gansu zokor, except that the percentage of the rectum microbiota obtained from the cecum was significantly different (*p* < 0.01) between species.

## 4. Discussion

### 4.1. The Suitable Habitat of the Two Zokor Species

Plateau zokors are mainly distributed throughout the QTP and Gansu zokors are mainly distributed throughout the Loess Plateau, with a sympatric distribution area in the transition zone in eastern QTP [35]. However, the distribution ranges of the two species and their sympatric area have not been studied nor defined clearly. Zhao et al. [36] believed that the altitude of 3100 m is a better criterion to distinguish the two species, and only the plateau zokor is distributed above the altitude of 3100 m. However, below the altitude of 3100 m, it is hard to determine the zokor species. In our study, based on the MaxEnt model [22,23], the suitable habitat and the overlap area of the two species were predicted clearly for the first time. The suitable habitat of the plateau zokors was mainly concentrated in eastern Qinghai and the southern Hexi Corridor in Gansu, while the Gansu zokors were mainly distributed in eastern Qinghai, the southern Hexi Corridor in Gansu, southern Ningxia and north central Shaanxi. The two zokor species overlapped in the area at the junction of eastern Qinghai and Gansu. Our results are consistent with the description of the distribution of different zokor species in China by Fan et al. [18]. Furthermore, our results will facilitate the study of population ecology of the plateau zokor and the Gansu zokor in the future.

### 4.2. The Difference of the Microbial Communities between Zokor Species and among Segments of the GIT

The characteristics of microbiota in different GIT segments of the two zokor species were first investigated in this study. We found that, for both species, the microbial community richness and diversity of the small intestine were almost the lowest while those of the cecum were the highest among the four segments of the GIT; the microbial community diversity of the small intestine in the plateau zokor was a little higher than that of the stomach. The high microbial community richness and diversity of the cecum is in accordance with the study on pikas by Lu et al. [37]. This may be a common feature for hindgut-fermenting herbivorous mammals because relatively higher bacterial diversity in the cecum—the major fermenting organ for hindgut-fermenting mammals—may provide favorable conditions for the fermentation of cellulose, starch, and other indigestible plant polysaccharides [17,25]. Moreover, a neutral pH environment in the cecum provides the possibility of high microbial diversity [17,38].

Additionally, beta diversity analyses for both the zokor species revealed that the gut region significantly influences the bacterial community structure, which is in accordance with the studies of Li et al. and Zhang et al. [17,25]. Differences among bacterial communities of different gut regions are likely related to several factors, such as pH, flow rate, nutrient supplies, immune responses, host secretions, and oxygen concentrations [17,39].

Gut microbiota are mainly influenced by host genetics and environmental factors [40], and food has frequently been reported to be the main factor [41,42]. When comparing the species in our study, the bacterial community compositions of the whole GIT, as well as each segment, were significantly different. There are two reasons for this. Firstly, species distributed in a sympatric area can gradually carve out a niche separation to reduce the interspecific competition for resources [42]. As for the two species investigated in this study, the plateau zokor prefers forbs, tuberous plants and even some highly toxic plants while the Gansu zokor prefers *Medicago sativa* and does not like any toxic plants [35]. Secondly, digestive tract morphology can affect digestive efficiency and probably determine an animal’s food list [43]. As reported, the length and structure of the small intestines of the two species are significantly different and the small intestine of the plateau zokor is much longer than that of the Gansu zokor [44]. This shows that the separation of diet and differences in digestive tract morphology may be the differentiating factors of bacterial community compositions between the two species. However, the diet and the digestive tract morphology are intrinsically influenced by the species identity, that is, the host’s genetics [9]. Thus, the differences in the bacterial community compositions of the whole GIT and each segment between the two zokor species may be due to host genetics. However, as typical subterranean and solitary rodents, the plateau and Gansu zokors spend most of their time in independent underground tunnels [15] and microbiota transfer between adult zokors can seldom happen except for some contact during the mating season. Thus, the maternal transmission should play a non-negligible role in the community composition of their gut microbiota, as well as the possible coprophagy happening within populations, which may also lead to the results observed in this study. To draw a definite conclusion on the role of host genetics compared to the maternal transmission or coprophagy, further studies including multiple sampling locations and multiple specimens per location would be needed.

It has always been controversial whether the bacterial community of the GIT can be represented by that of the feces. Studies on humans [45], bats [46], and blue mussels [47] have all demonstrated significant differences between the fecal and gut microbial communities. In this study, the bacterial community characteristics of different segments of the GIT, the stomach, small intestine, cecum, and rectum, are significantly different. Taking this into consideration, we believe that the bacterial community of feces cannot fully represent the profile of the GIT. For both the plateau zokor and the Gansu zokor, no significant differences were detected in the comparisons of the diversity and richness of the bacterial communities between the rectum and the cecum. What is more, as shown by the PCoA analysis, the bacterial community compositions of the rectum and the cecum were very similar. Although a significant difference in the bacterial community structure between the rectum and the cecum was found in the plateau zokor (*p* = 0.049), the difference was small, which can be inferred by the two very close circles representing the bacterial communities of the rectum and the cecum (Figure 3B). In the Gansu zokor, the bacterial community structures of the rectum and the cecum was not significantly different and even the same (Figure 3C). Thus, it was concluded that there is little difference between the bacterial communities of the cecum and the rectum and that the bacterial community of the cecum can be well represented by that of the rectum or the feces.

### 4.3. Indications of Source Tracking Analyses

The research on pika found that very little overlap occurred in the pika core bacteria and the most abundant soil bacteria [48]. However, underground life provides zokors more opportunities to be in contact with soil than ground animals. Does this mean that zokor’s gut microbiota are more vulnerable to soil microbiota? In this study, the source tracking analyses conducted on both zokors indicated that the soil has little effect on the bacterial community of the GIT and only the cG obtained more than 1% of the ASVs from the soil, which was greatly unexpected as we all know that both zokors are typical subterranean rodents and mainly feed on the underground roots and rhizomes of plants. This finding was consistent with that of Li et al. [48]. The most likely explanations for this surprising result could be that, first, zokors always peel the roots and rhizomes before eating them [49], which can obviously reduce the amount of the soil microorganisms ingested by feeding, and second, the high acid environment of the stomach can prevent most external microorganisms from entering and colonizing the GIT [50].

In our study, the highest percentages of the unknown source were detected in the cecum, with 70.39% in the plateau zokor and 67.54% in the Gansu zokor. This implies that the two species have formed a specific colonization pattern of microbes in their cecum that contribute to maintaining the ecosystem stability in this segment, and may provide favorable conditions for the fermentation of indigestible plant polysaccharides [17].

The source tracking analyses on the bacterial community of the stomach revealed pretty high percentages of the rectum source, 83.85% in the plateau zokor and 84.79% in the Gansu zokor. Based on these results, we guess that both zokors may perform the coprophagy behavior, which is a common phenomenon in small mammals [3,17,51]. For zokors, coprophagy may help to maintain the stability of the bacterial community of the GIT and nutrition in the feces released after the fermentation by hindgut microbiota can be ingested again, digested and absorbed through coprophagy. Moreover, the study conducted by Bo et al. [3] demonstrated that coprophagy can alter the cognitive ability of the Brandt’s vole (*Lasiopodomys brandtii*). However, due to the underground dwelling habit and limited indoor feeding experiment, evidence for coprophagy of the zokors from direct observation is still lacking and it remains to be studied what coprophagy means for zokors.

## 5. Conclusions

This was the first study conducted on the microbial biogeography along the GIT segments of the plateau zokor and the Gansu zokor distributed in a sympatric area. We found that the gut region significantly influences the bacterial community structure of the two species. For the bacterial community compositions of the whole GIT, as well as each segment, the conspecific difference of the bacterial community was smaller than the heterospecific differences. Source tracking analysis conducted on both zokors indicated that the soil has little effect on the bacterial community of the GIT. A fairly high percentage of rectum source in the bacterial community of the stomach indicated that both zokors may engage in coprophagy. However, it remains to be studied what coprophagy means for zokors. Our results provide important information for further study on the function of microbiota communities in different GIT segments and the potential use of the gut microbiota as a new method for the population management of zokors.

## Figures and Tables

**Table 1:** The numbers of different microbiological taxonomic units in this study.

Taxonomic Units	Plateau Zokor			Gansu Zokor			Total
	GIT (Gastrointestinal Tract)	Tunnel Soil	Share	GIT	Tunnel Soil	Share	
Phylum	15	36	12	11	34	10	39
Class	29	104	21	21	96	15	117
Order	75	224	49	53	207	28	271
Family	123	332	72	81	320	35	429
Genus	255	582	101	159	584	39	872
ASV(amplicon sequence variant)	4319	4774	101	3646	4568	28	15,046

## Data Availability

Sequence data are available from the Sequence Read Archive (SRA) BioProject PRJNA749684.
